# Comparative Metabolite Profiling of Wild and Cultivated *Justicia procumbens* L. Based on ^1^H-NMR Spectroscopy and HPLC-DAD Analysis

**DOI:** 10.3390/plants9070860

**Published:** 2020-07-07

**Authors:** Hyunyong Lee, Jihyun Jeon, Joobyoung Yoon, Seung-Hwan Kim, Hyun Sik Choi, Jong Seung Kang, Yong Sup Lee, Mase Lee, Young Ho Kim, Hwan Bong Chang

**Affiliations:** 1 Research Institute, Dong Wha Pharmaceutical Company, Yongin-si, Gyeonggi-do 17084, Korea; hyunyong.lee@dong-wha.co.kr (H.L.); jihyun.jeon@dong-wha.co.kr (J.J.); joobyoung.yoon@dong-wha.co.kr (J.Y.); seunghwan.kim@dong-wha.co.kr (S.-H.K.); hyunsik.choi@dong-wha.co.kr (H.S.C.); mase.lee@dong-wha.co.kr (M.L.); 2 College of Pharmacy, Chungnam National University, Daejeon 34134, Korea; kangjss@cnu.ac.kr; 3 Department of Life and Nanopharmaceutical Sciences, Kyung Hee University, Seoul 02453, Korea; kyslee@khu.ac.kr

**Keywords:** *Justicia procumbens*, DW2008S, NMR fingerprint, OPLS-DA, justicidin B, diphyllin

## Abstract

*Justicia procumbens* L. is known across Korea, India, China, and Taiwan as a remedy against fever, cough, sore throat, and cirrhosis of ascites. *J. procumbens* provides the raw material for a candidate anti-asthma drug (DW2008S) currently completing phase I clinical trials sponsored by Dong Wha Pharmaceutical Company. HPLC-DAD was used to quantify phytochemical constituents of *J. procumbens*, and HPLC and ^1^H-NMR results were assessed by multivariate analysis. This is the first time a comparative study using HPLC-DAD and NMR fingerprints has been applied to identify chemical differences between wild and cultivated *J. procumbens*. The amount of justicidin B as the marker compound was higher in cultivated samples (0.80 ± 0.25 mg/g) than in wild ones (0.63 ± 0.30 mg/g). Orthogonal partial least squares discriminant analysis (OPLS-DA) from HPLC and NMR data revealed that there were clear differences between wild and cultivated types and identified five secondary metabolites, which could help distinguish between wild and cultivated plants. Among these five lignans, diphyllin showed the most potent discrimination between two types and was significantly detected higher in cultivated ones than in wild ones. A combination of ^1^H-NMR and HPLC-DAD analysis is effective for *J. procumbens* standardization and metabolomics studies.

## 1. Introduction

*Justicia procumbens* L. (known in English as Oriental water willow) is a plant belonging to the Acanthaceae family and is widely distributed in Korea, India, Taiwan, and Southern China. In Korea, one species of *Justicia* is listed as Jwi-kko-ri-mang-cho [1]. The entire *J. procumbens* plant is commonly used in Korea, China, and India in traditional medicine for the treatment of fever, cough, edema, jaundice, sore throat, and urinary tract infection [2]. The aerial part of *J. procumbens* is reported as an edible plant in the Korean Food Code [3].

The powdered ethanol extract of *J. procumbens* (DW2008S) is currently completing phase I clinical trials and is ready to proceed to phase II as a candidate against asthma. The extract has been shown to effectively improve Th_2_-driven airway inflammation and bronchoconstriction by inhibiting T-cell immunoreceptor with Ig and ITIM domains, phosphodiesterase 4, and A_3_ adenosine receptor activities [4,5].

Previous studies have reported that various lignans—such as justicidins A–D, tuberculatin, taiwanin E, taiwanin E methyl ether, neojusticin A, neojusticin B, chinensinaphthol, and pronaphthalide A—exhibit anti-tumor, anti-viral, anti-platelet aggregation, and anti-inflammatory activities [6,7,8,9]. Justicidin B, diphyllin, and phyllamyricin C isolated from *Phyllanthus polyphyllus* L. inhibit tumor necrosis factor-α and nitric oxide production in lipopolysaccharide/interferon-γ-activated peritoneal macrophages; whereas justicidin A has been reported to inhibit the growth of human colorectal HT-29 and HCT 116 cancer cells [10,11,12,13]. High-performance liquid chromatography (HPLC) fingerprint profiling has been used previously for identification and quality control of the active components obtained from *J. procumbens* [14,15,16,17].

Recently, proton nuclear magnetic resonance (^1^H-NMR) profiling enabled the simultaneous detection of abundant primary metabolites (organic acids, amino acids, and carbohydrates) as well less abundant secondary metabolites (terpenes, flavonoids, alkaloids, and lignans) typically found in plants [18,19,20,21].

In addition, as the cultivation of medicinal plants is gaining increasing momentum for the purpose of conserving natural plant resources and facilitating access to active constituents, many comparative studies of wild and cultivated plants have been reported [22,23]. A standardized quality of herbal products is an essential step towards the cultivation of wild plants.

At present, there is no report on using multivariate analysis such as principal component analysis (PCA), hierarchical clustering analysis (HCA), and orthogonal partial least squares discriminant analysis (OPLS-DA) to compare wild and cultivated *J. procumbens*. Therefore, in this study, a systematic comparative method based on multivariate analysis of HPLC and ^1^H-NMR profiles was set up to compare cultivated and indigenous Korean *J. procumbens*. The aim of the study was to standardize quality control of wild and cultivated Korean *J. procumbens.*

## 2. Results

### 2.1. HPLC Analysis and Peak Identification

**Figure 1:**
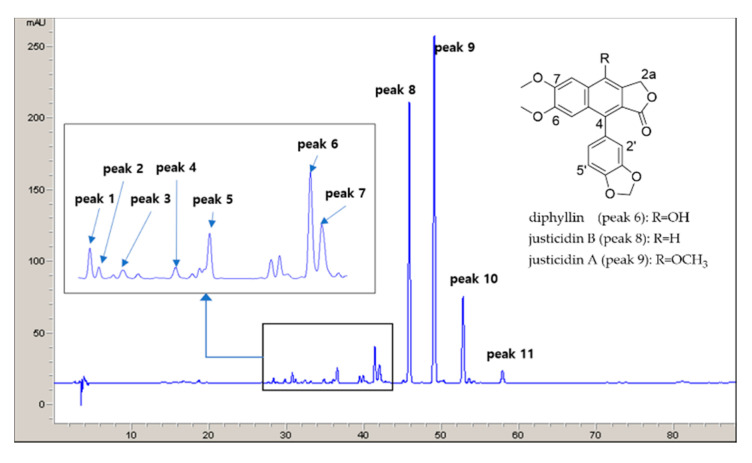
HPLC chromatogram of *J. procumbens*.

To analyze the relationship and intraspecific variations between the abundance of lignan compounds and different collection types, we first selected 11 common peaks with >1% of total peak area within 30–55 min of retention time in 35 samples of *J. procumbens*. The detected peaks were identified by comparing their retention times, UV spectra, and mass spectrometry (MS) data to those of pure compounds. Their structure was determined by comparing MS and NMR spectral data with published data for azizin (compound 1, peak 1) [24], ciliatoside B (compound 2, peak 2) [25], justicidinoside A (compound 3, peak 3) [7], tuberculatin (compound 4, peak 4) [26], 6′-hydroxyjusticidin B (compound 5, peak 5) [27], diphyllin (compound 6, peak 6) [28,29], 2a-hydroxyjusticidin A (compound 7, peak 7) [30], justicidin B (compound 8, peak 8) [12,29,31], justicidin A (compound 9, peak 9) [29,31,32], justicidin C (compound 10, peak 10, overlap of compound 11) [33], phyllamyricin C (compound 11, peak 10, overlap of compound 10) [34], and neojusticin A (compound 12, peak 11) [27,32] (Table S1, Figure 1 and Figure S1).

Justicidin B was selected as a marker compound because it was detected consistently and strongly in all 35 chromatograms (Figure S2). Chemically synthesized justicidin B has been used as the standard marker for quality control of *J. procumbens*, and its various biological activities and chemical synthesis have already been described [12,35,36].

Based on the validated HPLC method (Tables S1, S2, S3, and S4), the content of justicidin B in dried *J. procumbens* was estimated to range from 0.22 to 1.38 mg/g (*w/w*) (Table 1).

### 2.2. ^1^H-NMR Analysis

**Figure 2:**
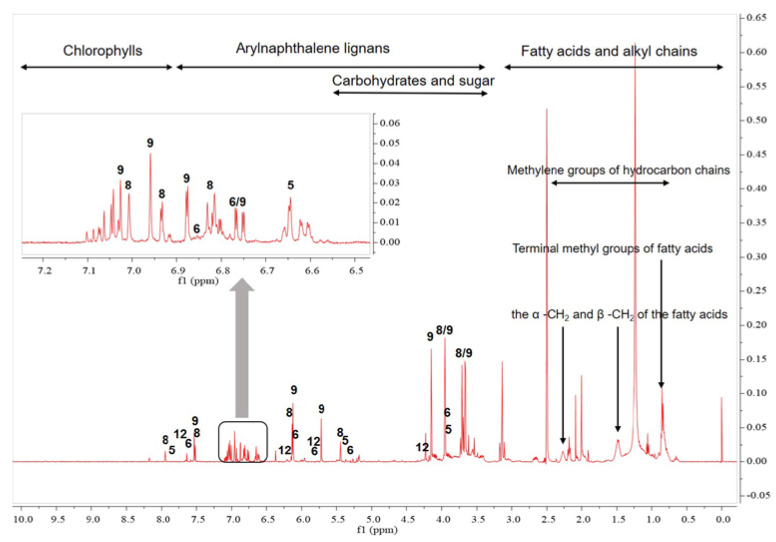
Representative ^1^H-NMR spectrum of *J. procumbens.* Peak 5: 6′-hydroxyjusticidin B; Peak 6: diphyllin; Peak 8: justicidin B; Peak 9: justicidin A; Peak 12: neojusticin A.

**Figure 3:**
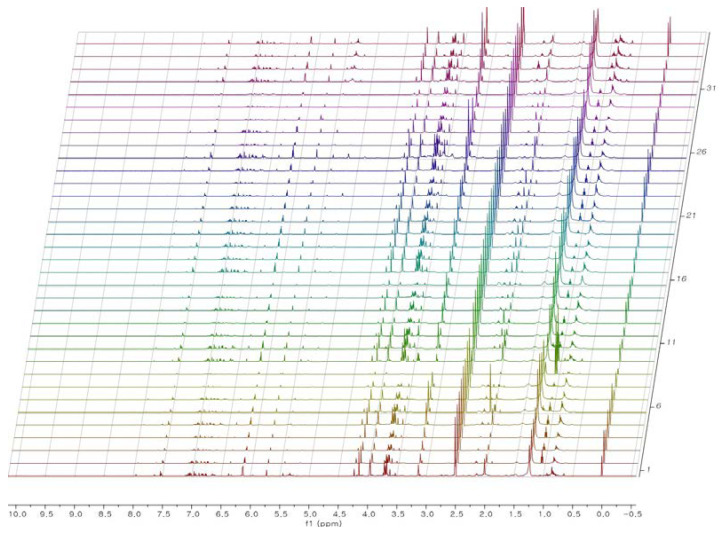
Stacked ^1^H-NMR spectra of *J. procumbens* (*n* = 35).

^1^H-NMR analysis provided useful information about the chemical constituents of *J. procumbens* (Figure 2 and Figure 3). Fatty acids constituted the predominant peaks in the upfield region of the spectra. The presence of saturated and unsaturated fatty acids was evidenced by the characteristic signals of terminal methyl (0.85 and 0.93 ppm), different methylene groups of the hydrocarbon chain (1.17 to 2.2 ppm), and olefinic protons (5.32 ppm) [18,19].

The mid-region (3.0–5.5 ppm) of the spectra, denoting primarily the characteristic peaks of carbohydrates and sugars, displayed an abundance of carbohydrate ring protons [20,21]. The strong singlet peaks at 3.6–4.2 ppm were contributed by the methoxy groups of arylnaphthalene lignans at positions C-1, 6, and 7 (Figure 1 and Figure S1). The region at 5.5–8.0 ppm showed the aromatic proton peaks contributed by arylnaphtalene lignans and phenolic compounds [24,25,26,27,28,29,30,31,32,33,34,35,36]. The low-field region of the spectrum (8.0–10.0 ppm) was characterized mainly by peaks denoting chlorophyll components and was excluded from this multivariate analysis because it was weaker than the other regions [18,37,38]. As with HPLC, ^1^H-NMR analysis confirmed that justicidin B (compound 8) and justicidin A (compound 9) were the major metabolites present in *J. procumbens* (Figure 1, Figure 2, Figure 3 and Figure S3).

### 2.3. Multivariate Analysis

Secondary metabolites in plants vary considerably due to numerous factors that affect their biosynthetic pathways and accumulation. These factors can be environmental, genetic, onto-genic, and morphogenetic [39,40,41]. Not surprisingly, plants of the same species grown in distinct environments may exhibit different concentrations of a particular secondary metabolite.

PCA identifies new PCs using only independent variables and regression equations, which is particularly useful for detecting outliers. Group separation, however, is not always observable in PCA models because changes originating from various pathological states may be small compared to other types of intra- and inter-sample variations. In contrast, OPLS-DA identifies new variables by considering both dependent as well as independent variables and uses them to find a regression equation. As a regression method, OPLS-DA uses spectroscopic data and specific properties of the data to correlate each group with its data matrix. It allows easy determination of the associated variation between observations and different groups by rotating the axis [42,43].

The aim of coupling HPLC-DAD with ^1^H-NMR analysis was to identify specific metabolites that were most informative about the above factors and easy to distinguish between wild and cultivated samples.

#### 2.3.1. Multivariate Analysis of HPLC-DAD Data

**Figure 4:**
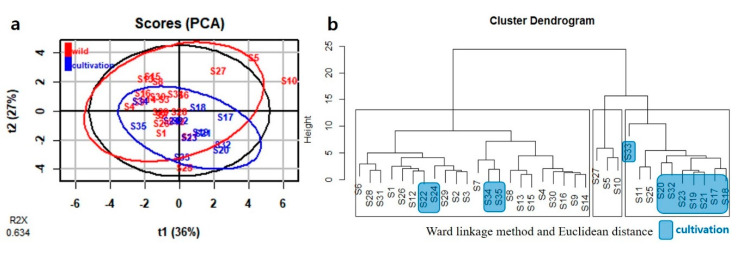
PCA and HCA of HPLC data in *J. procumbens.* (**a**) PCA; (**b**) HCA.

PCA was performed using as variable eleven peak areas obtained from HPLC analysis. The first two PCs explained 63.4% of the total variance: 36.5% by PC1 and 26.9% by PC2 (Figure 4a). HCA was performed next. The Ward linkage method was applied, and Euclidean distance was selected as a measure. Samples were grouped into three clear groups (solid-line boxes in Figure 4b). Twenty-two samples, 10 samples, and 3 samples were grouped into groups I, II, and III, respectively (Figure 4b). Based on PCA and HCA, nine cultivated samples were located in the part with positive values for PC1. Compared to them, 16 wild samples were located in the part with negative values for PC1 (Figure 4a). PC1 correlated positively with all peaks except peak 6 (Figure S4a).

To further investigate the potential constituents of two groups (wild and cultivated) in 11 peaks, samples from two groups were subjected to OPLS-DA in R studio using the R ‘ropls’ package. The fitting quality and reliability of (O)PLS-DA models were verified by cross-validation and *p*-value using quality parameters R^2^X, R^2^Y, and Q^2^Y, as well as permutation tests R^2^X and R^2^Y are the values of determination for the X and Y matrices, and Q^2^Y is a measure of the predictive capability of the model based on cross-validation [44,45].

**Figure 5:**
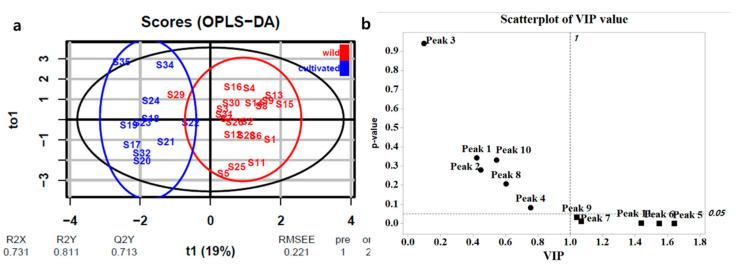
Identification of HPLC-DAD data components crucial for sample classification. (**a**) OPLS-DA score plot of HPLC data; (**b**) VIP values.

Preliminary analysis of the data pointed to three outliers (S10, S27, and S33 in Figure S5) representing uninteresting operating conditions, so these were excluded from further consideration [44,45]. The samples were divided into two groups (wild and cultivated) in the score plot and the R^2^X, R^2^Y, and Q^2^Y values of fitting goodness were 0.731, 0.811, and 0.713, respectively (Figure 5a). To identify the components of HPLC-DAD data crucial for sample classification, variable importance in projection (VIP) scores were used. Peaks with VIP scores >1 were deemed important for differentiating between wild and cultivated types [40,41]. Five such markers were identified as peaks 5 (6′-hydroxyjusticidin B, VIP score: 1.64), 6 (diphyllin, VIP score: 1.55), 7 (2a-hydroxyjusticidin A, VIP score: 1.07), 9 (justicidin A, VIP score: 1.04), and 11 (neojusticin A, VIP score: 1.44) with significant *p*-values (<0.05) as shown in Figure 5b.

**Figure 6:**
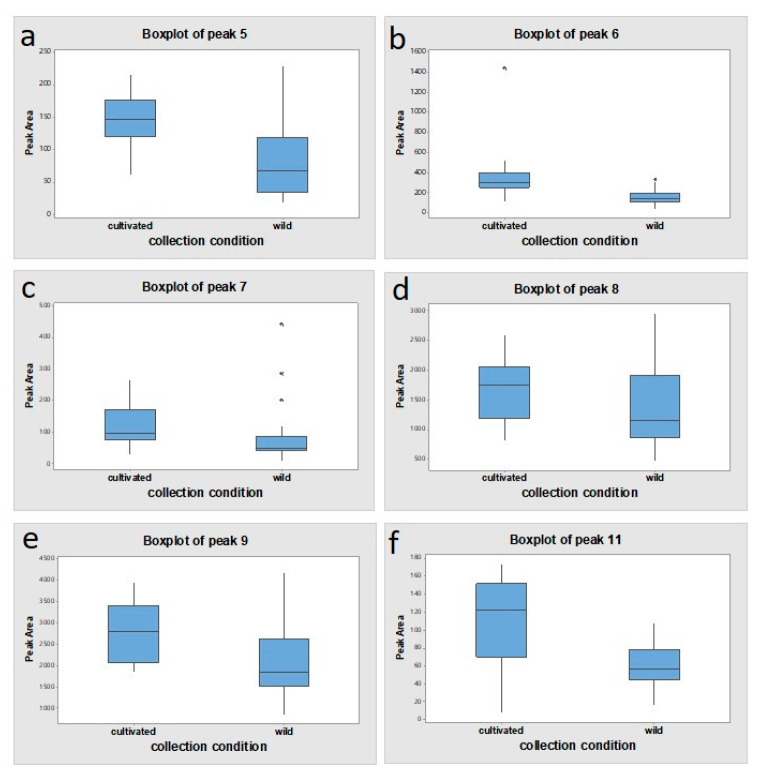
HPLC peak area comparison of peaks 5, 6, 7, 8, 9, and 11 between wild and cultivated collection types. (**a**) 6′-hydroxyjusticidin B *; (**b**) diphyllin *; (**c**) 2a-hydroxyjusticidin A *; (**d**) justicidin B; (**e**) justicidin A *; (**f**) neojusticin A *, * *p* < 0.05; cultivated type vs. wild type.

As shown in Figure 6, the mean peak areas of these five lignans were larger in cultivated samples than in wild samples with significant *p*-values (<0.05). As well as, the amount of justicidin B as the marker compound was higher in cultivated samples (0.80 ± 0.25 mg/g) than in wild ones (0.63 ± 0.30 mg/g).

#### 2.3.2. Multivariate Analysis of ^1^H-NMR Data

**Figure 7:**
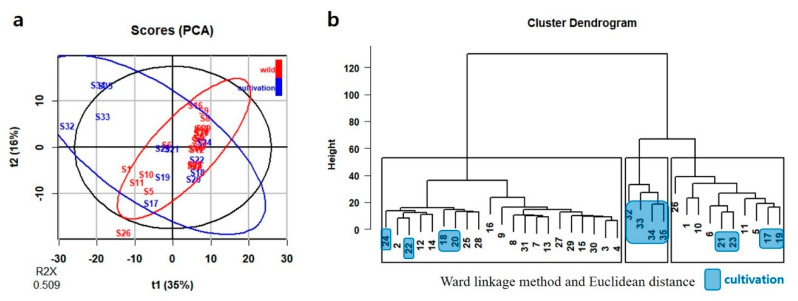
PCA and HCA of ^1^H-NMR data in *J. procumbens* (*n* = 35). (**a**) PCA; (**b**) HCA.

Figure 7a provides an overview of the localized NMR data that can be discerned by an unsupervised PCA. The first two PCs cumulatively accounted for 50.9% of the total variance: 34.9% by PC1 and 16.0% by PC2 (Figure 7a). HCA cluster dendrogram showed three clusters of the 35 samples following the Ward linkage method and Euclidean distance measurement (solid-line boxes in Figure 7b). Twenty-one samples, 10 samples, and 4 samples were grouped into groups I, II, and III, respectively (Figure 7b). Based on PCA and HCA, eight cultivated samples were located in the part with negative values for PC1. Compared to them, 17 wild samples were located in the part with positive values for PC1 (Figure 7a). The correlation between PC1 and PC2 were showed in the loading plot of PC1 and PC2, and more than half of the variables were shown positive values for PC1 (Figure S4b).

**Figure 8:**
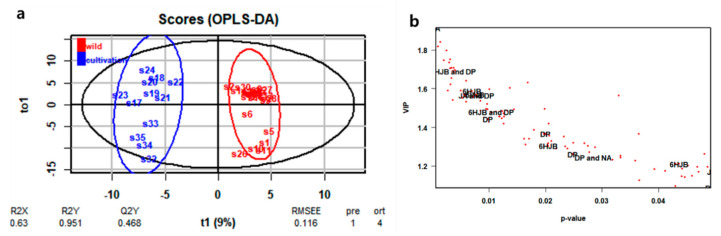
Identification of ^1^H-NMR data components crucial for sample classification. (**a**) OPLS-DA score plot of ^1^H-NMR data; (**b**) VIP values (>1.0, *p* < 0.05). 6HJB: 6′-hydroxyjusticidin B; DP: diphyllin; JA: justicidin A; NA: neojusticin A; FA: fatty acids.

The PCA and HCA of ^1^H-NMR spectral data failed to show a clear classification with respect to cultivation location and collection type. Supervised OPLS-DA was carried out to diagnose the latent metabolic variations that had led to the discrete patterns observed by PCA. Two groups of data were submitted to OPLS-DA: wild type and cultivated type. OPLS-DA score plots based on ^1^H-NMR analysis of wild and cultivated types from 35 samples showed clear separation between wild and cultivated types (Figure 8a). The samples were divided into two groups (wild and cultivation) in the score plot, and the R^2^X, R^2^Y, and Q^2^Y values of fitting goodness were 0.63, 0.951, and 0.468, respectively (Figure 8a).

According to the OPLS-DA results, 98 variables from binned set data were selected as significant markers based on their VIP score (>1.0) with significant *p*-values (<0.05). As shown in Figure 8b, the VIP scores plot of OPLS-DA revealed that arylnaphthalene lignans and fatty acids were the major contributors to the classification between wild and cultivation types, partially confirming HPLC-DAD results.

Several ^1^H-NMR peaks of these significant markers were identified as arylnaphthalene lignans in comparison to reference compounds: 6′-hydroxyjusticidin B (7.89–7.91, 7.47–7.49, 6.95–6.97, 6.01–6.03, and 5.41–5.43 ppm), diphyllin (6.85–6.87, 6.09–6.11, and 5.75–5.77 ppm), justicidin A (7.53–7.55 and 6.73–6.77 ppm), and neojusticin A (7.63–7.65, 6.21–6.23, 5.75–5.77, and 3.39–3.41 ppm) (Figure 2, Figure S3 and Table S6).

## 3. Discussion

A disadvantage of herbal medicines is the poor characterization of active constituents. The composition of these compounds can vary greatly from batch to batch, depending on the weather, geographic location, collection type (wild or cultivated), collection time, and extraction procedure. To overcome this limitation, it is important to characterize the chemical structure and composition of bioactive compounds in herbal medicines. Given the increasing interest in quality control of products containing plant raw materials, HPLC and ^1^H-NMR pattern analysis has become an important tool for medicinal plant identification and standardization. Chromatography spectrometry and NMR spectroscopy have become the main methods for profiling and quantifying plant metabolite compositions. NMR techniques are highly reproducible, provide valuable structural information, and represent an attractive alternative to targeted chromatographic analysis.

In the present study, PCA and HCA on HPLC and ^1^H-NMR results failed to provide a clear relationship between domestic geographic origin and chemical composition. Environmental factors appear to have only a minor effect on chemical diversity between wild populations of *J. procumbens* in Korea. Also, there was no similarity in cluster dendrograms of HCA based on HPLC and ^1^H-NMR results. It could be that these different cluster dendrograms were contributed from differences between the targeted approach (HPLC analysis) and the non-targeted approach (NMR analysis) methods.

However, score plots of OPLS-DA (Figure 5 and Figure 8) built using both ^1^H-NMR and HPLC-DAD data showed a clear differentiation between wild and cultivated samples, with R^2^X > 0.6 suggesting a marked metabolomic difference among these samples. Based on HPLC and NMR analysis, these differences were ascribed to several lignans and several primary metabolites, and especially diphyllin (compound 6) showed the most potent discrimination between wild and cultivated samples. Moreover, diphyllin was equally in the VIP top three of both OPLS-DA models, which suggests the importance of the biosynthesis pathways related to justicidin B as a marker compound in *J. procumbens* [12]. Also, this result showed that environmental growth conditions influenced the production of plant metabolites.

Interestingly, the results clearly show that the metabolites under investigation as potential anti-asthma drugs are more abundant in cultivated *J. procumbens* than in wild samples. Moreover, this characteristic makes them candidate markers for rapidly discriminating between the two collection types.

## 4. Materials and Methods

### 4.1. Plant Material Collection and Cultivation

A total of 35 samples were collected over a period of eight years (2012–2019) from different locations in Korea. As shown in Table S5, 12 samples were cultivated at several sites (Jecheon-si, Yongin-si, Yeongdong-gun, and Yangpyeong-gun). In particular, five samples grown in Jecheon-si obtained the good agricultural practices (GAP) certification from the Chungbuk Technopark Korea Medicine & Natural Products Center, Cheongju-si, Chungbuk, Korea.

All specimens were deposited at Dong Wha Pharmaceutical Company and carefully authenticated by Professor Young Ho Kim (College of Pharmacy, Chungnam National University, Daejeon, Korea). Cultivated specimens were deposited at the National Institute of Biological Resources (NIBR), Ministry of Environment, Incheon, Korea, and certificated by Dr. Jin Seok Kim (NIBR) based on morphological characteristics.

### 4.2. Sample Preparation

Crude dried plant samples were ground and 2.0 g of sample powder was extracted by ultrasonication with 50 mL of anhydrous ethanol for 20 min at room temperature. After centrifugation at 3000 rpm (1948× *g*, radius 19.3 cm, Gyrozen^®^ 1580; Gyrozen, Gimpo, Korea) for 5 min, the supernatant was filtered through filter paper (5 µm; Advantec MTF Inc., Dublin, CA, USA). The residue was extracted by ultrasonication with 40 mL of anhydrous ethanol for 10 min at room temperature, then centrifuged and filtered as above. The filtrates were combined into a final volume of 100 mL and passed through a syringe filter (0.2 µm, PTF Whatman; Sigma-Aldrich, St. Louis, MO, USA) for HPLC analysis.

The above ethanol extract (10 mL) was concentrated in a vacuum oven at 65 °C for 24 h and reconstituted in DMSO-*d_6_* (1 mL) with 0.03% (*v/v*) tetramethylsilane (TMS; Sigma-Aldrich) as the internal frequency lock (0.00 ppm). The dissolved plant extracts were vortex for 1 min at room temperature (20–25 °C) and centrifuged at 15,000× *g* (Micro 17R; Hanil Scientific Inc., Incheon, Korea) at room temperature for 10 min using a microtube to obtain a clear supernatant for NMR experiments.

### 4.3. HPLC-DAD Analysis and ^1^H NMR Analysis

#### 4.3.1. HPLC-DAD Analysis

HPLC-DAD analysis was carried out on a Shiseido (Tokyo, Japan) Capcell Pak UG120 C18 column (4.6 × 250 mm, 5 µm); the column temperature was set to 35 °C. The mobile phase consisted of (A) acetonitrile and (B) water at a flow rate of 0.8 mL/min, with gradient elution as follows: 0-5 min, 15% A; 40 min, 46% A; 60 min, 55% A; 70 min, 60% A (*v/v*); 75 min, 40% A; 76-90 min, 15% A *(v/v).* Details are explained in the section on method validation in Supplementary Materials.

#### 4.3.2. ^1^H-NMR Spectra Acquisition

^1^H-NMR spectra were acquired on a JEOL ECA 500 MHz spectrometer (JEOL Ltd., Tokyo, Japan). The supernatants (550 µL) were transferred to 5-mm NMR tubes and analyzed. Each spectrum was acquired with 128 scans, a spectral width of 15 ppm, and a constant temperature of 25 °C. Prior to statistical analysis, all ^1^H-NMR spectra were referenced to 0.03% TMS, automatically phased, baseline corrected (Whittaker smoother), subjected to removal of the solvent signals (DMSO-*d*_6_), and desaturated at the water peak.

### 4.4. Multivariate Data Processing

The HPLC peak areas were post-processed by mean-centering as the default setting in R Studio (ver. 1.2.5042). HPLC peak area boxplot comparisons were performed in Minitab (ver. 18.1) (Minitab LLC., State College, PA, USA) to compared wild samples with cultivated samples. The NMR data were post-processed by median method normalization and Pareto scaling for a better elucidation of the compounds contributing to the separation of each plot in the score plots, and converted to ASCII files using MestReNova 11.0 (Mestrelab Research, Santiago de Campostela, Spain). Spectra in the 0.00–10.00 ppm range were blinded by the solvent peak (2.55 ppm, DMSO-*d*_6_) and the region of 8.00–10.00 ppm was characterized by chlorophyll constituents. The water peak (3.3 ppm) was removed by pre-saturation. The signal was integrated into bins of 0.02 ppm in width, resulting in 285 variables. The generated ASCII files were first imported into Microsoft Excel (version 2019) for secondary variable labeling, and then into R Studio for PCA, HCA, and OPLS-DA. The whole data set was used without splitting off a test set for cross-validation in OPLS-DA.

## 5. Conclusions

Based on multivariate analysis, such as PCA and OPLS-DA, the specific five lignan compounds isolated from *J. procumbens* have been identified as suitable biomarkers to discriminate between different collection types. A combination of HPLC and ^1^H-NMR analysis offers the ability to discern between *J. procumbens* indigenous to Korea and its cultivated species. The analysis confirmed the stable composition of justicidin B, further supporting its selection as a marker compound for quality control of Korean *J. procumbens*.

In summary, the methods described herein appear suitable for the standardization of plant raw materials based on Korean *J. procumbens* and could be extended to the standardization of herbal drugs. Furthermore, this study would promote the cultivation of this species for its raw material and contribute to the standardization and production of herbal medicines from *Justicia* species.

## Figures and Tables

**Table 1:** Justicidin B content in *J. procumbens*.

Sample No.	Content (mg/g, *n* = 3)	Sample No.	Content (mg/g, *n* = 3)	Sample No.	Content (mg/g, *n* = 3)
S1	1.00 ± 0.04	S13	0.38 ± 0.03	S25	1.38 ± 0.08
S2	0.67 ± 0.00	S14	0.45 ± 0.10	S26	0.89 ± 0.09
S3	0.49 ± 0.01	S15	0.33 ± 0.09	S27	0.40 ± 0.02
S4	0.40 ± 0.01	S16	0.28 ± 0.02	S28	0.97 ± 0.23
S5	0.54 ± 0.12	S17 ^#^	0.86 ± 0.11	S29	0.46 ± 0.04
S6	0.58 ± 0.04	S18 ^#^	0.54 ± 0.07	S30	0.33 ± 0.02
S7	0.95 ± 0.04	S19 ^#^	0.79 ± 0.08	S31	0.59 ± 0.03
S8	0.22 ± 0.04	S20 ^#^	1.21 ± 0.04	S32 ^#^	1.12 ± 0.02
S9	0.51 ± 0.07	S21 ^#^	0.88 ± 0.02	S33 ^#^	1.03 ± 0.08
S10	0.74 ± 0.19	S22 ^#^	0.75 ± 0.03	S34 ^#^	0.40 ± 0.02
S11	1.13 ± 0.33	S23 ^#^	0.84 ± 0.02	S35 ^#^	0.64 ± 0.12
S12	0.69 ± 0.08	S24 ^#^	0.53 ± 0.02	-	-

^#^ cultivated samples.

## Supplementary Materials

Experimental data, as well as supplementary Tables S1–S6, and Figure S1–S4, are available online at https://www.mdpi.com/2223-7747/9/7/860/s1. Figure S1: Structure of compounds (1–12) isolated from *Justicia procumbens*; Figure S2: HPLC chromatogram of *J. procumbens* (*n* = 35); Figure S3: Stacked ^1^H-NMR spectra of reference compounds and *J. procumbens* extract; Figure S4: Loading score plots of variables in PCA. (a) HPLC-DAD data; (b) 1H-NMR data; Figure S5: OPLS-DA model of the collection type response in *J. procumbens* (*n* = 35). Blue: cultivated plants (*n* = 12); red: wild plants (*n* = 23). (**a**) x-score plot showing the number of components and cumulative R^2^X, R^2^Y, and Q^2^Y values (*n* = 35); (**b**) Outlier diagnostics of samples; Table S1: Chromatographic, UV, and mass spectrometry (MS) data of peaks 1~11 analyzed by HPLC-DAD; Table S2: Calculated parameters for the calibration curve (*n* = 3); Table S3: Repeatability (intra-day, *n* = 6) and intermediate precision (inter-day, *n* = 3) of justicidin B; Table S4: Recovery of justicidin B; Table S5: Samples used in this study; Table S6: Summary of VIP values (6HJB: 6’-hydroxyjusticidin B; DP: diphyllin; JA: justicidin A; NA: neojusticin A; FA: fatty acids).
