# Supplementary material for: Comparative Metabolite Profiling of Wild and Cultivated Justicia procumbens L. Based on 1H-NMR Spectroscopy and HPLC-DAD Analysis
Source: Plants (Basel). 2020 Jul 7;9(7):860. doi: 10.3390/plants9070860 (PMC7411763; doi:10.3390/plants9070860)
Supplement: Supplementary file 1 [file plants-09-00860-s001.pdf]

Supplementary Materials

Method validation

HPLC method was used for determination of the content of marker compound (Justicidin B) in *Justicia procumbens*. To demonstrate the method's suitability for its intended purpose, the method was validated by parameters such as specificity, linearity, precision, accuracy, limit of quantification (LOQ), limit of detection (LOD), and system suitability.

Specificity and system suitability

Specificity was evaluated by identifying any interferences in retention time of justicidin B from the blank and sample solution. System suitability was evaluated by determining the relative standard deviation (RSD) of measured peak areas in six repeat injections of the standard solution (50 μg/mL).

No interferences in retention time of justicidin B from the blank and sample solution was observed. The retention time of justicidin B was confirmed to be 47.3 min. System suitability was deemed good, with 0.97% RSD for the justicidin B peak area.

Linearity and range

Linearity was evaluated by the coefficient of determination (R^2) of the calibration curve. To construct the calibration curve, a stock solution of justicidin B standard was prepared at 1000 μg/mL with N,N-dimethyl sulfoxide (50 mg/g per sample); this solution was then diluted to eight different concentrations (0.125~5 mg/g) with anhydrous ethanol. The calibration curve solution was prepared in triplicate for each concentration. The calibration curves were constructed by plotting peak areas vs. concentration of each calibration curve solution.

As shown in Table S2, the linear regression equation was y = 2140.0x + 57.7 and the calibration curve was good in the range 0.125~5 mg/g (R^2 = 0.9999), thus confirming the linearity of justicidin B.

LOD and LOQ

Based on the calibration curves, the LOD and LOQ were estimated from the standard deviation of the response and the slope. They were estimated as 3.3 σ/S and 10 σ/S, respectively, where σ is the standard deviation of y-intercepts of the regression equation and S is the slope. The LOQ was verified by evaluating the signal-to-noise (S/N) ratio and recovery of six samples prepared at the LOQ concentration.

The LOD and LOQ calculated from the linear regression equation were 0.017 mg/g and 0.051 mg/g, respectively. The S/N ratio of LOQ was 49.2 and recovery was 102.5% (2.04% RSD, n = 6). Thus, the LOQ was verified (Table S2).

Precision and accuracy

Repeatability (intra-day) and intermediate precision (inter-day) were evaluated by determining the RSD of the measured amount of justicidin B in six sample solutions for three days. The effect of random events was confirmed by changing days, analysts, HPLC systems, and columns. Accuracy was

evaluated by determining the recovery of samples spiked with three different concentrations of justicidin B (10, 30, and 50 μg/mL) in sample solution.

Repeatability and intermediate precision were good, as indicated by 0.84~1.88% intra-day RSD and 1.41% inter-day RSD, respectively (Table S3). Recovery was also good (100.5%, 2.80% RSD) (Table 3). Thus, precision and accuracy were confirmed.

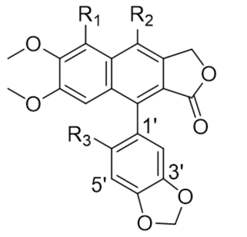

1 R_1 = H, R_2 = *O*-[*β*-D-xylopyranosyl-(1′′′→2′′)-*β*-D-xylopyranosyl-(1′′′′→5′′)]-*β*-D-apiofuranosyl, R_3 = H
2 R_1 = H, R_2 = *O*-[*β*-D-apiofuranosyl-(1′′′′′→ 3′′′)-*α*-L-arabinopyranosyl-(1′′′→2′′)][*β*-D-xylopyranosyl-(1′′′′→5′′)]-*β*-D-apiofuranosyl, R_3 = H
3 R_1 = H, R_2 = OCH_3, R_3 = O-*β*-D-glucopyranosyl
4 R_1 = H, R_2 = O-*β*-D-apiofuransyl, R_3 = H
5 R_1 = H, R_2 = H, R_3 = OH
6 R_1 = H, R_2 = OH, R_3 = H
8 R_1 = H, R_2 = H, R_3 = H
9 R_1 = H, R_2 = OCH_3, R_3 = H
11 R_1 = OCH_3, R_2 = H, R_3 = H

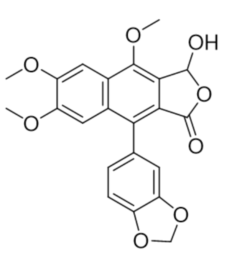

7

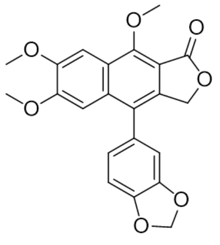

10

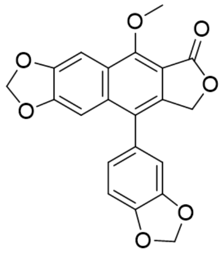

12

Figure S1. Structure of compounds (1–12) isolated from *Justicia procumbens*.

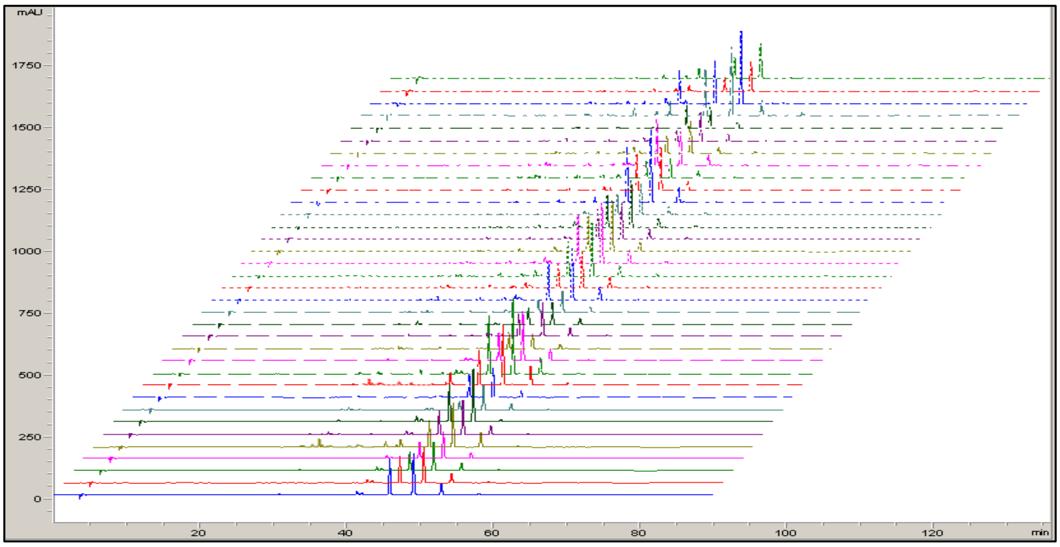

Figure S2. HPLC chromatogram of *J. procumbens* (n = 35).

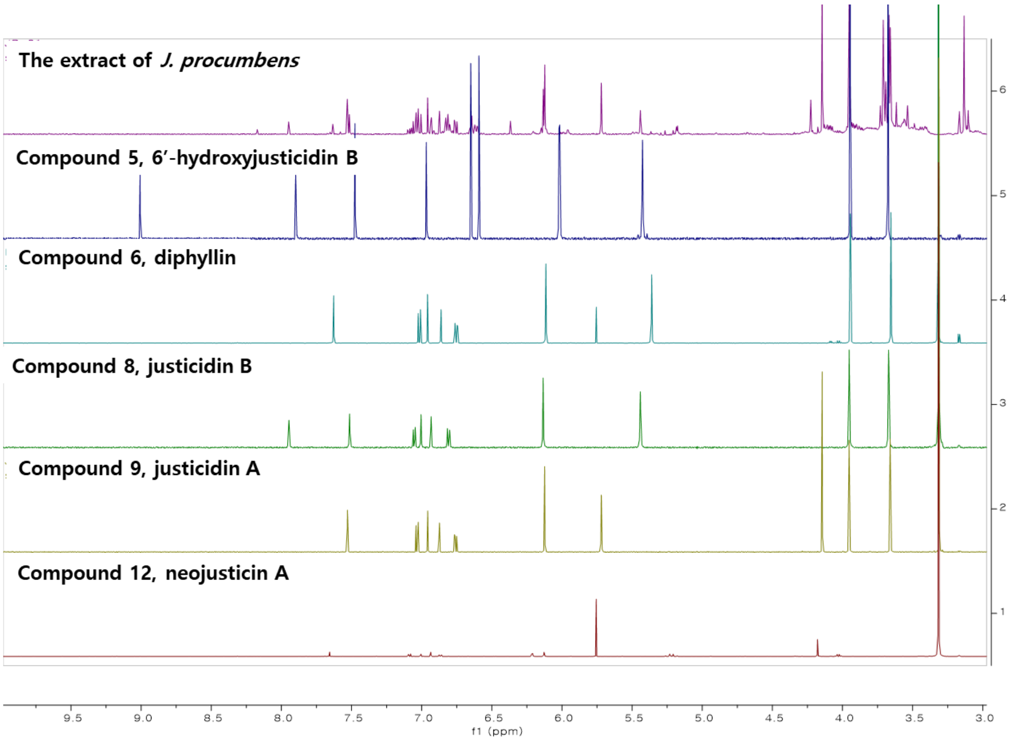

Figure S3. Stacked ^{1}H-NMR spectra of reference compounds and *J. procumbens* extract.

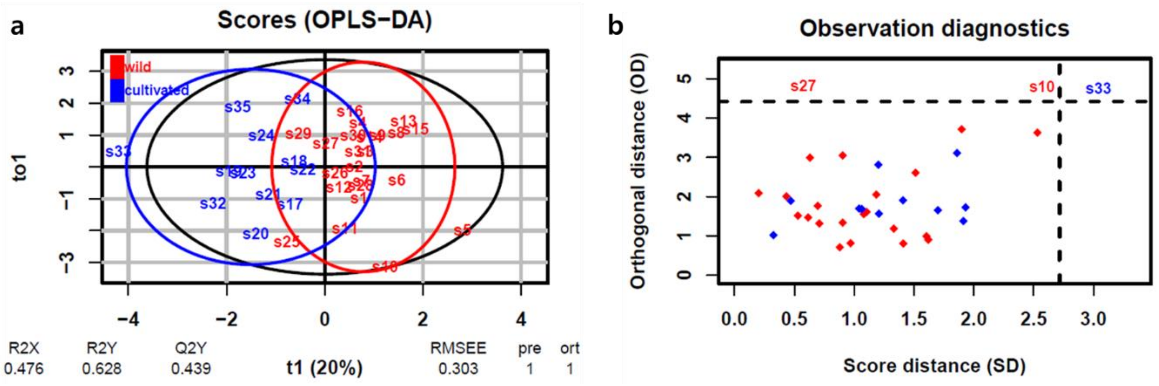

Figure S4. OPLS-DA model of the collection type response in *J. procumbens* (n = 35). Blue: cultivated plants (n = 12); red: wild plants (n = 23). (**a**) x-score plot showing the number of components and cumulative R^2X, R^2Y, and Q^2Y values (n = 35); (**b**) Outlier diagnostics of samples.

Table S1. Chromatographic, UV, and mass spectrometry (MS) data of peaks 1~11 analyzed by HPLC-DAD.

Peak no.	t_R (min)	Compound name	λmax (nm)	MS
1	30.7	azizin	201, 228, 262, 315	645.2 $[M+H]^+$
2	31.2	ciliatoside B	201, 228, 262, 315	931.2 $[M+Na]^+$
3	32.3	justicidinoside A	201, 222, 262, 315	595.2 $[M+Na]^+$
4	34.8	tuberculatin	201, 229, 262	513.2 $[M+H]^+$
5	36.5	6′-hydroxyjusticidin B	201, 222, 258, 310	381.1 $[M+H]^+$
6	41.3	diphyllin	201, 231, 268	381.1 $[M+H]^+$
7	41.9	2a-hydroxyjusticidin A	201, 262	411.1 $[M+H]^+$
8	45.8	justicidin B	201, 223, 258, 296, 310	365.1 $[M+H]^+$
9	49.0	justicidin A	201, 234, 262, 310	395.1 $[M+H]^+$
10	52.8	overlap of justicidin C[a] and phyllamyricinC[b]	201, 262, 318	[a] 395.1 $[M+H]^+$<br>[b] 395.1 $[M+H]^+$
11	57.8	neojusticin A	201, 262, 318	379.1 $[M+H]^+$

t_R: retention time.

Table S2. Calculated parameters for the calibration curve (n = 3).

Compound	Regression equation	R^2	Range (mg/g)	LOD (μg/g)	LOQ (μg/g)
justicidin B	Y = 2140.0 x + 57.7	0.9999	0.125~5	0.017	0.051

Equation: y = peak area, x = concentration of standard (mg/g); LOD: limit of detection; LOQ: limit of quantification; R^2: coefficient of determination.

Table S3. Repeatability (intra-day, n = 6) and intermediate precision (inter-day, n = 3) of justicidin B.

Sample no.	Intra-day		Inter-day	
	Mean ± SD	RSD (%)	Mean ± SD	RSD (%)

S20	1.22 ± 0.01	0.84	1.21 ± 0.02	1.41
	1.20 ± 0.02	1.88		
	1.21 ± 0.01	1.00		

RSD: relative standard deviation; SD: standard deviation.

Table S4. Recovery of justicidin B.

Recovery sample (Conc.)	Q1*	Q2**	Q3 (n = 3)***	Recovery (%)	Total rec. (%)	RSD (%)
justicidin B (10 μg/mL)	23.72	9.86	33.96 ± 0.06	103.9 ± 0.73	100.5 ± 2.81	2.80
justicidin B (30 μg/mL)	23.72	29.57	53.16 ± 0.42	99.6 ± 1.74		
justicidin B (50 μg/mL)	23.72	49.28	71.99 ± 0.05	98.0 ± 0.12		

*Q1: quantity of justicidin B (μg/mL) in unspiked sample solution (S20); **Q2: quantity of spiking standard solution (μg/mL, "added"); ***Q3: quantity of justicidin B (μg/mL) in spiked sample solution; RSD: relative standard deviation.

Table S5. Samples used in this study.

No.	Collection site	Collection date	Collection type
S1	Yeongdeok-dong, Giheung-gu, Yongin-si, Gyeonggi-do	2012.10	Wild
S2	Sampyeong-dong, Bundang-gu, Seongnam-si, Gyeonggi-do	2013.8	Wild
S3	Jayang-dong, Dong-gu, Daejeon	2013.9	Wild
S4	Yeongdeok-dong, Giheung-gu, Yongin-si, Gyeonggi-do	2013.9	Wild
S5	Ha-dong, Yeongtong-gu, Suwon-si, Gyeonggi-do	2013.10	Wild
S6	Makgye-dong, Gwacheon-si, Gyeonggi-do	2013.10	Wild
S7	Idong-eup, Cheoin-gu, Yongin-si, Gyeonggi-do	2013.10	Wild
S8	Myeonmok-dong, Jungnang-gu, Seoul	2013.10	Wild
S9	Ha-dong, Yeongtong-gu, Suwon-si, Gyeonggi-do	2013.10	Wild
S10	Yeongdeok-dong, Giheung-gu, Yongin-si, Gyeonggi-do	2013.10	Wild
S11	Bora-dong, Giheung-gu, Yongin-si, Gyeonggi-do	2013.10	Wild
S12	Gongse-dong, Giheung-gu, Yongin-si, Gyeonggi-do	2013.10	Wild
S13	Gongse-dong, Giheung-gu, Yongin-si, Gyeonggi-do	2013.10	Wild
S14	Gongse-dong, Giheung-gu, Yongin-si, Gyeonggi-do	2013.11	Wild
S15	Jigok-dong, Giheung-gu, Yongin-si, Gyeonggi-do	2013.11	Wild
S16	Sajeong-dong, Jung-gu, Daejeon	2013.11	Wild
S17	Gongse-dong, Giheung-gu, Yongin-si, Gyeonggi-do	2014.9	Cultivation

S18	Gongse-dong, Giheung-gu, Yongin-si, Gyeonggi-do	2015.9	Cultivation
S19	Korea National Arboretum, Damun-ri, Yongmun-myeon, Yangpyeong-gun, Gyeonggi-do	2015.9	Cultivation
S20	Gangje-dong, Jecheon-si, Chungcheongbuk-do	2015.9	Cultivation
S21	Korea National Arboretum, Damun-ri, Yongmun-myeon, Yangpyeong-gun, Gyeonggi-do	2016.9	Cultivation
S22	Gangje-dong, Jecheon-si, Chungcheongbuk-do	2016.9	Cultivation
S23	Dohwa-ro, Songhak-myeon, Jecheon-si, Chungcheongbuk-do	2017.9	Cultivation
S24	Dohwa-ro, Songhak-myeon, Jecheon-si, Chungcheongbuk-do	2018.9	Cultivation
S25	Songhak-myeon, Jecheon-si, Chungcheongbuk-do	2018.11	Wild
S26	Dongnam-ro, Yangnam-myeon, Gyeongju-si, Gyeongsangbuk-do	2014.10	Wild
S27	Banpo-daero, Seocho-gu, Seoul	2014.10	Wild
S28	Makgye-dong, Gwacheon-si, Gyeonggi-do	2014.10	Wild
S29	Gyo-dong, Wansan-gu, Jeonju-si, Jeollabuk-do	2015.8	Wild
S30	Gung-dong, Yuseong-gu, Daejeon	2017.9	Wild
S31	Inpung-ri, Jeongan-myeon, Gongju-si, Chungcheongnam-do	2017.10	Wild
S32	Gangje-dong, Jecheon-si, Chungcheongbuk-do	2019.9	Cultivation
S33	Hangok-ri, Yongsan-myeon, Yeongdong-gun, Chungcheongbuk-do	2017.10	Cultivation
S34	Hangok-ri, Yongsan-myeon, Yeongdong-gun, Chungcheongbuk-do	2018.10	Cultivation
S35	Hangok-ri, Yongsan-myeon, Yeongdong-gun, Chungcheongbuk-do	2019.10	Cultivation
